# Supplementary figures and images for: Posterior circulation acute stroke prognosis early CT scores in predicting functional outcomes: A meta-analysis
Source: PLoS One. 2021 Feb 16;16(2):e0246906. doi: 10.1371/journal.pone.0246906 (PMC7886215; doi:10.1371/journal.pone.0246906)

# Unfavorable outcome prediction by PC-ASPECTS per score decrease (varied imaging modality)

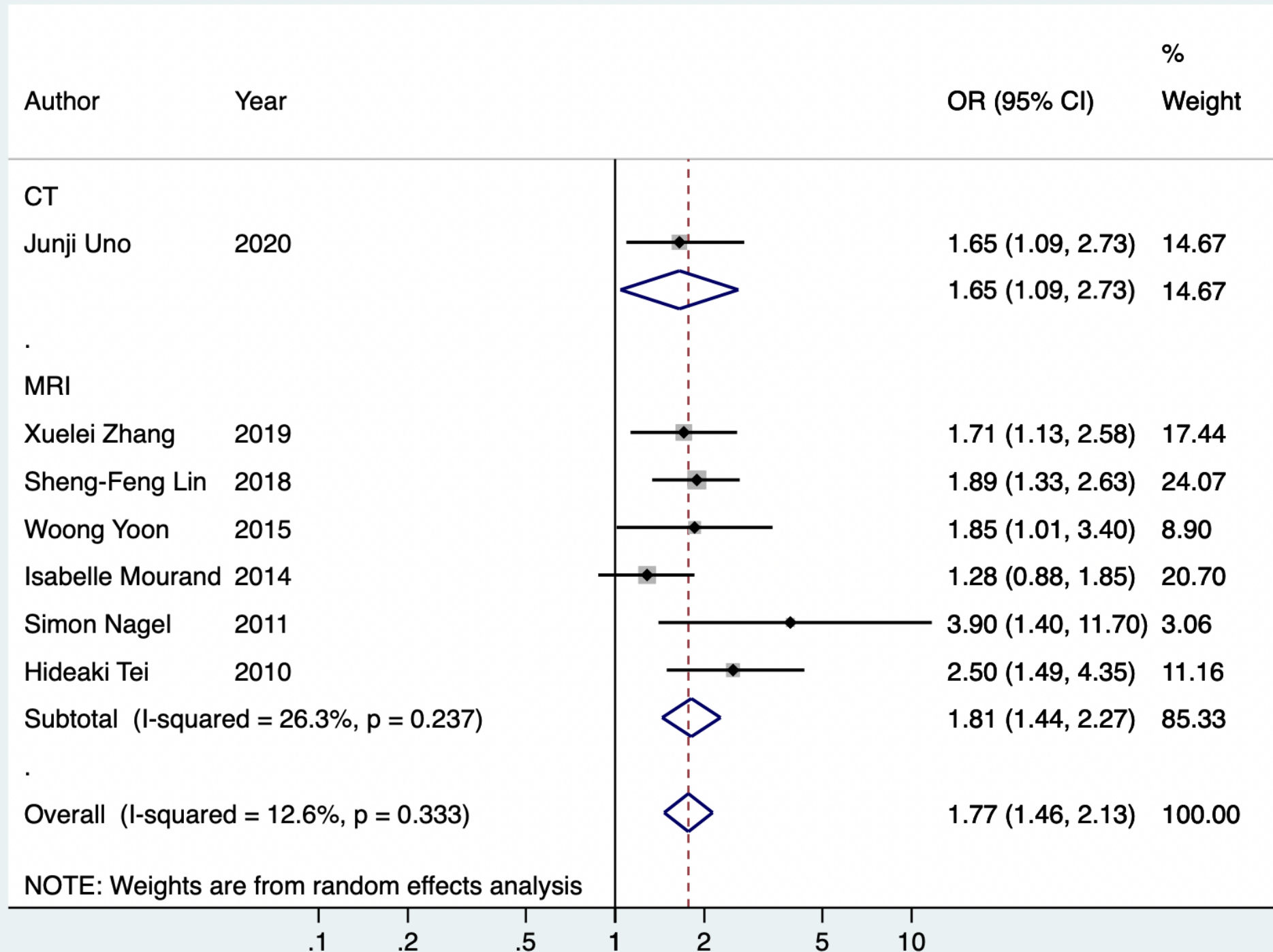

Supplement: S1 Fig — (PDF) [file pone.0246906.s002.pdf]

# Unfavorable outcomes prediction with binary PC-ASPECTS (varied imaging modality)

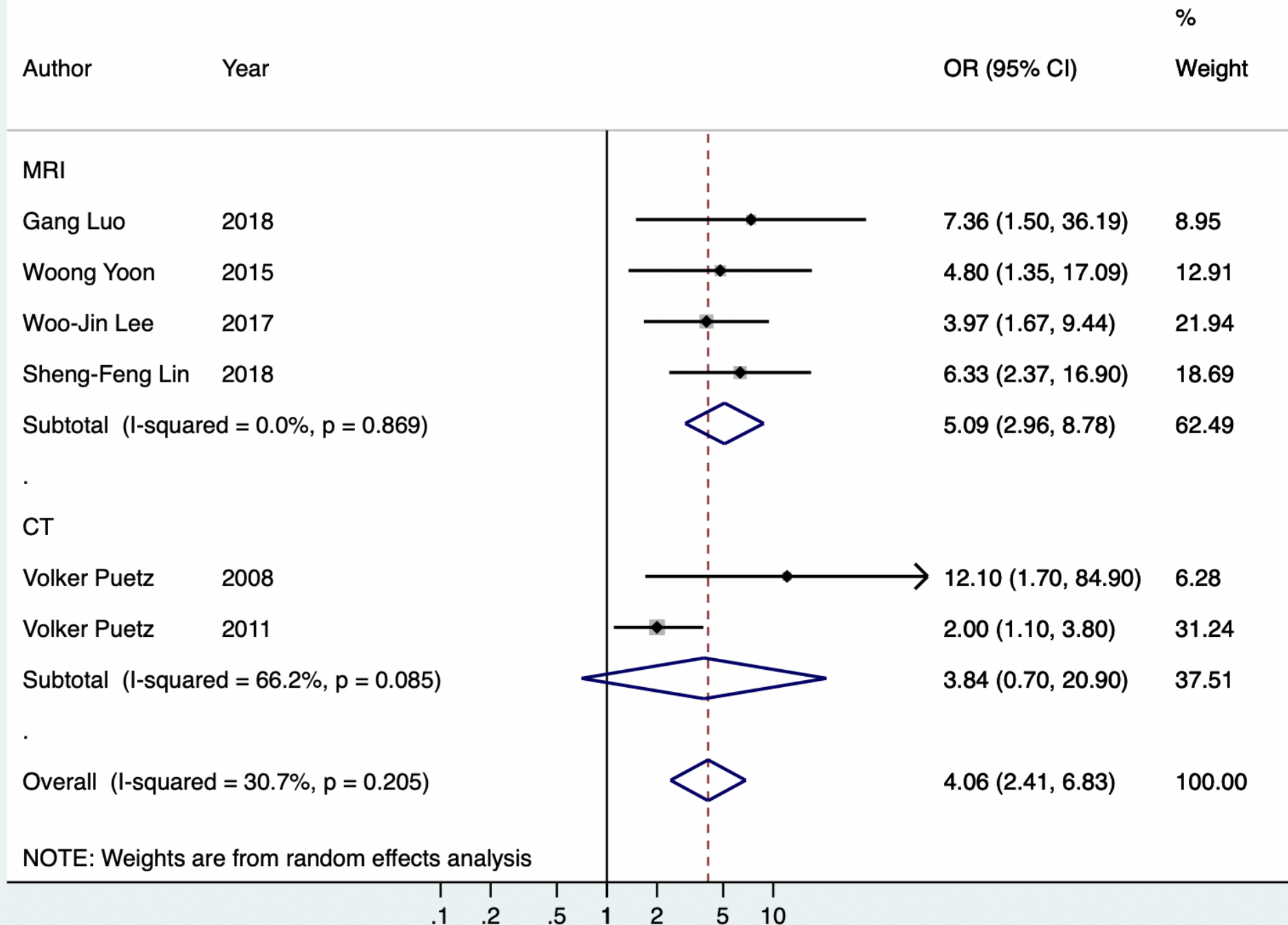

Supplement: S2 Fig — (PDF) [file pone.0246906.s003.pdf]

# PC-ASPECTS score difference (varied imaging modality)

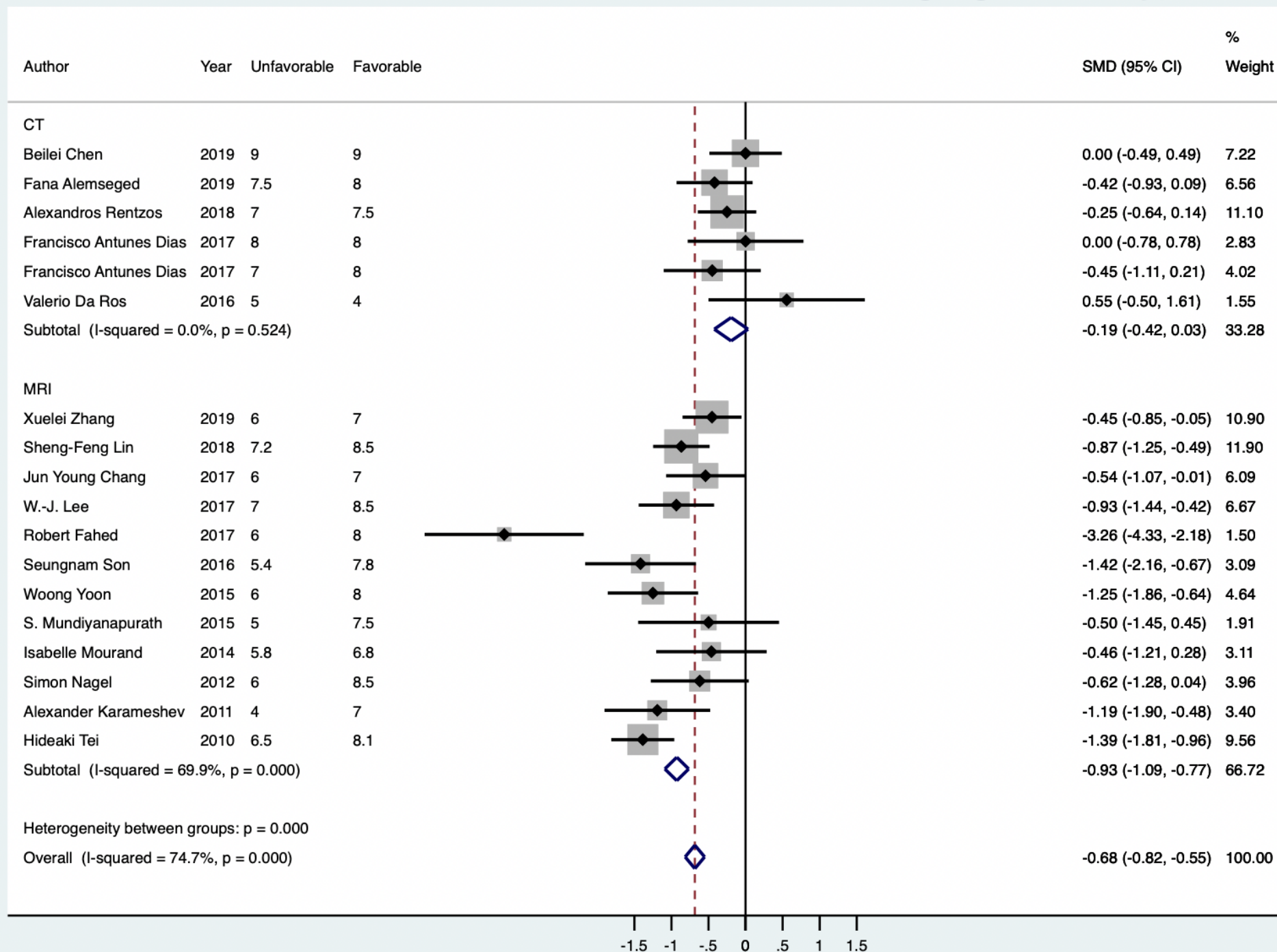

Supplement: S3 Fig — (PDF) [file pone.0246906.s004.pdf]

# Unfavorable outcome prediction by PC-ASPECTS per score decrease (varied definition of mRS)

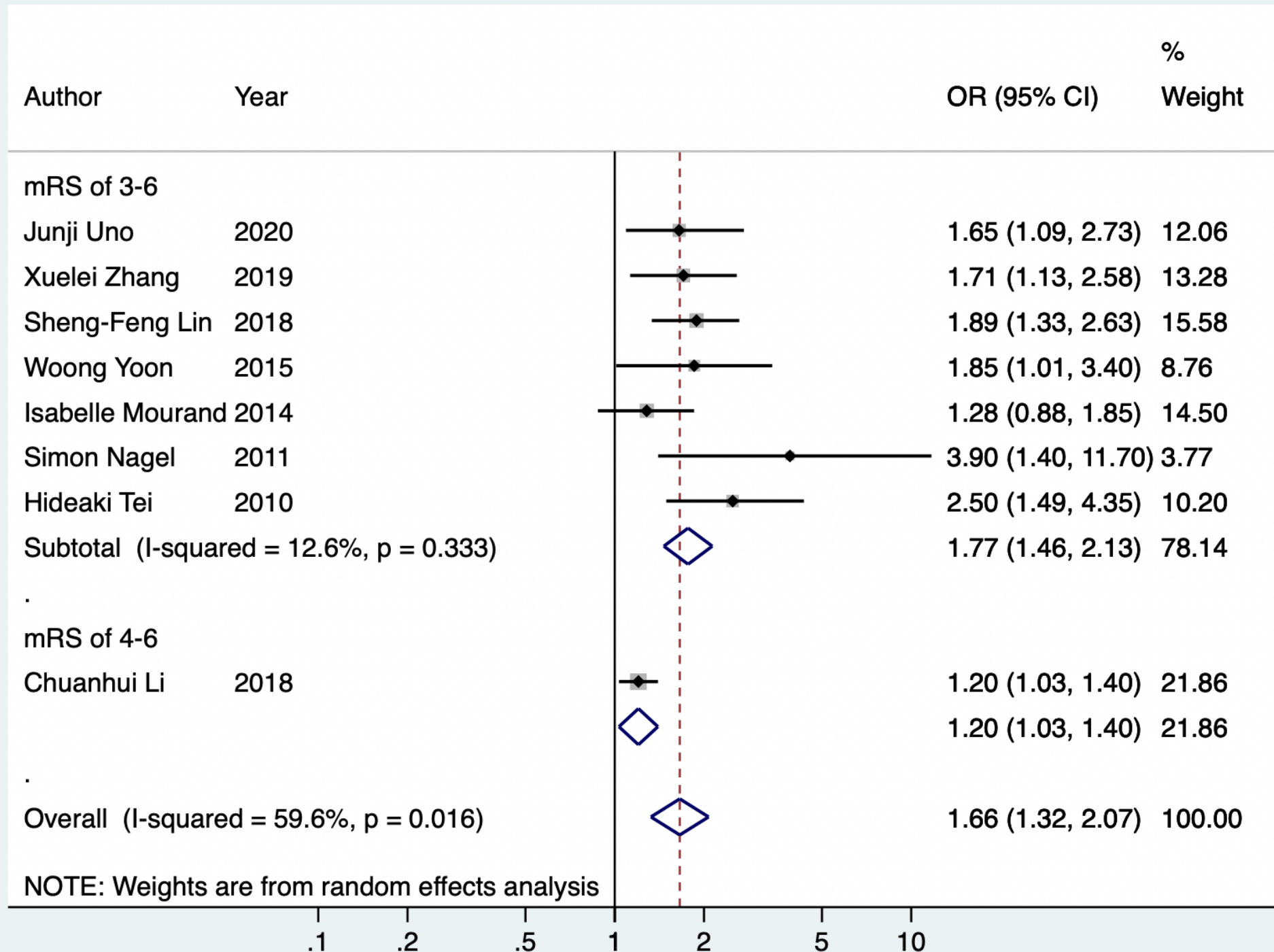

Supplement: S4 Fig — (PDF) [file pone.0246906.s005.pdf]

# Unfavorable outcomes prediction by binary PC-ASPECTS (varied definition of mRS)

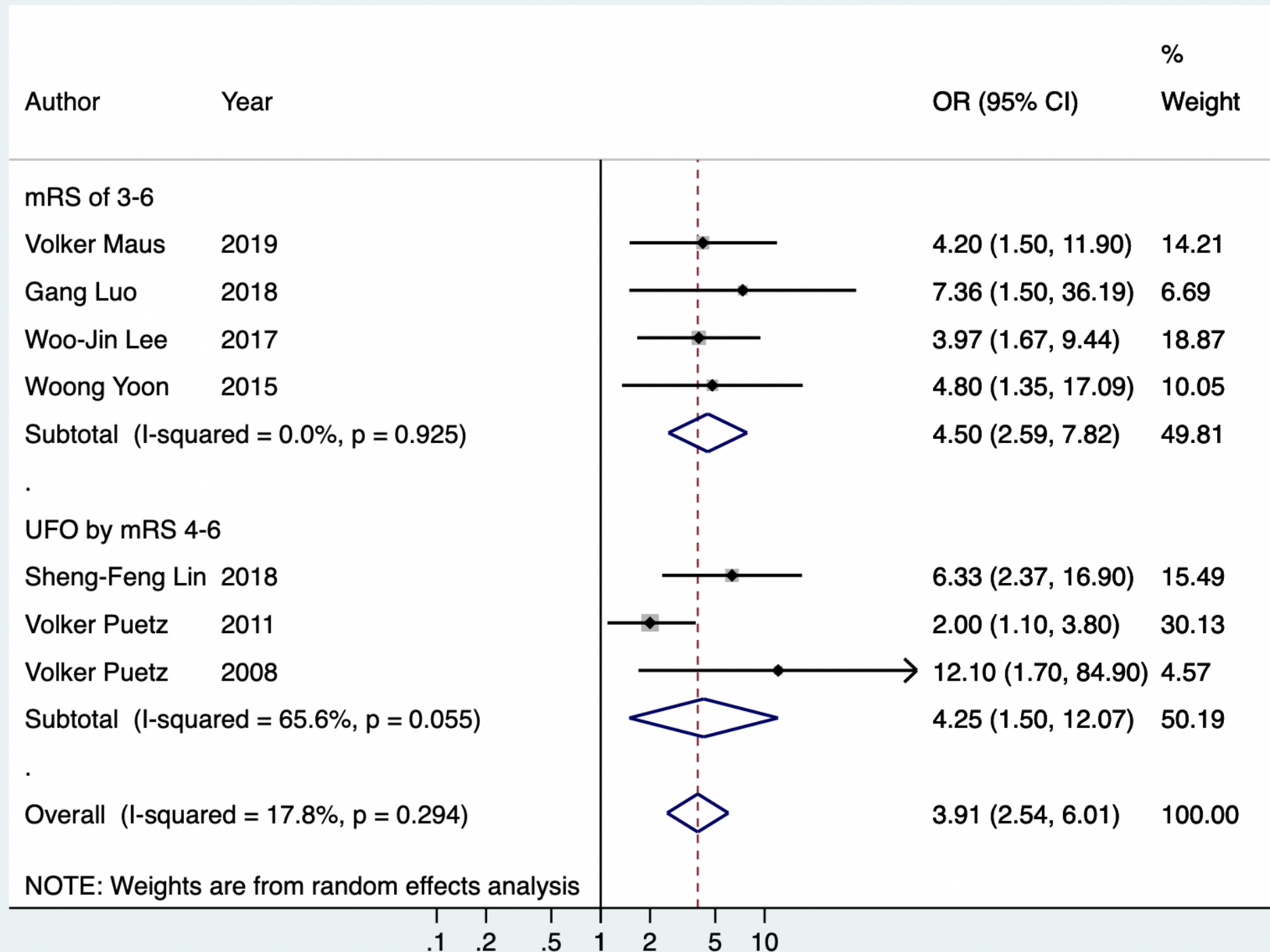

Supplement: S5 Fig — (PDF) [file pone.0246906.s006.pdf]

# PC-ASPECTS score difference (varied definition of mRS)

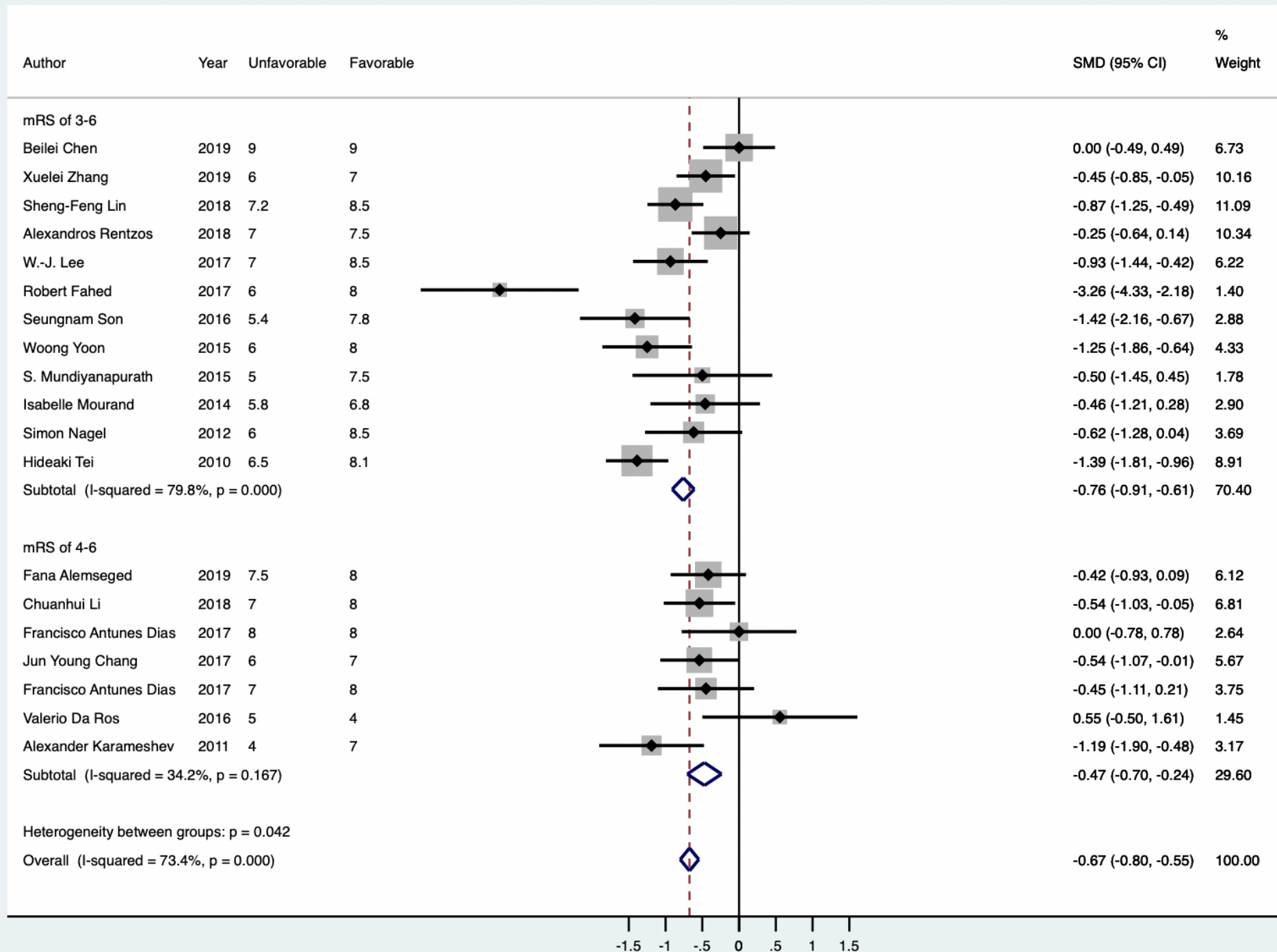

Supplement: S6 Fig — (PDF) [file pone.0246906.s007.pdf]
