# Supplementary material for: Posterior circulation acute stroke prognosis early CT scores in predicting functional outcomes: A meta-analysis
Source: PLoS One. 2021 Feb 16;16(2):e0246906. doi: 10.1371/journal.pone.0246906 (PMC7886215; doi:10.1371/journal.pone.0246906)
Supplement: S1 Table — (PDF) [file pone.0246906.s008.pdf]

S1 Table. Search strategy.

**PubMed search accessed on 1 Aug. 2020 (25 studies of 2488 results)**	**Embase search accessed on 1 Aug. 2020 (8 studies of 1945 results)**	**Scopus search accessed on 1 Aug. 2020 (0 studies of 12 results)**
Keywords: ((Stroke) AND (posterior circulation) AND (ASPECTS)) OR ((Stroke) AND (PC-ASPECTS)) OR ((Cerebrovascular disorders) AND (basilar artery) AND ((thrombolytic therapy)) OR ((Cerebrovascular disorders) AND (basilar artery) AND ((endovascular procedures)) OR ((Cerebrovascular disorders) AND (vertebrobasilar insufficiency) AND ((endovascular procedures)) OR ((Stroke) AND (vertebrobasilar insufficiency) AND ((endovascular procedures)) OR (Alberta stroke program early CT score) OR ((basilar artery) AND (thrombolytic therapy)) OR ((vertebrobasilar insufficiency) AND (thrombolytic therapy)) OR ((vertebrobasilar insufficiency) AND (endovascular procedures)) OR ((basilar artery) AND (endovascular procedures))		
