# Supplementary material for: Posterior circulation acute stroke prognosis early CT scores in predicting functional outcomes: A meta-analysis
Source: PLoS One. 2021 Feb 16;16(2):e0246906. doi: 10.1371/journal.pone.0246906 (PMC7886215; doi:10.1371/journal.pone.0246906)
Supplement: S2 Table — (PDF) [file pone.0246906.s009.pdf]

S2 Table. Quality measure of included studies by the Newcastle-Ottawa quality assessment scale

Study Name	Selection				Comparability		Exposure			Total
	1)	2)	3)	4)	a)	b)	1)	2)	3)	
**Scale: Favourable vs unfavourable PC-ASPECTS**										
Beilei Chen 2019	*	*	*	*		*	*	*	*	8
Xuelei Zhang 2019	*	*	*	*		*	*	*	*	8
Fana Alemseged 2019	*	*	*	*		*	*	*	*	8
Alexandros Rentzos 2018	*	*	*	*		*	*	*	*	8
Chuanhui Li 2018	*	*	*	*		*	*	*	*	8
Sheng-Feng Lin 2018	*	*	*	*		*	*	*	*	8
Jun Young Chang 2017	*	*	*	*		*	*	*	*	8
Francisco Antunes Dias 2017	*	*	*	*		*	*	*	*	8
W.-J. Lee 2017	*	*	*	*		*	*	*	*	8
Francisco Antunes Dias 2017	*	*	*	*		*	*	*	*	8
Robert Fahed 2017	*	*	*	*		*	*	*	*	8
Seungnam Son 2016	*	*	*	*		*	*	*	*	8
Valerio Da Ros 2016	*	*		*		*	*	*	*	7
Woong Yoon 2015	*	*	*	*		*	*	*	*	8
S. Mundiyanapurath 2015	*	*	*	*		*	*	*	*	8
Isabelle Mourand 2014	*	*	*	*		*	*	*	*	8
Simon Nagel 2012	*	*	*	*		*	*	*	*	8
Alexander Karameshev 2011	*	*	*	*		*	*	*	*	8
Hideaki Tei 2010	*	*	*	*		*	*	*	*	8
**Study Name**	**Selection**				**Comparability**		**Outcome**			**Total**
	1)	2)	3)	4)	a)	b)	1)	2)	3)	
**Scale: PC-ASPECTS discriminating functional outcomes**										
Volker Maus 2019	*	*	*	*		*	*	*	*	8
Sheng-Feng Lin 2018	*	*	*	*		*	*	*	*	8
Gang Luo 2018	*	*	*	*		*	*	*	*	8
Woo-Jin Lee 2017	*	*	*	*		*	*	*	*	8
Woong Yoon 2015	*	*	*	*		*	*	*	*	8
Volker Puetz 2011	*	*	*	*		*	*	*	*	8
Volker Puetz 2008	*	*	*	*		*	*	*	*	8
**Study Name**	**Selection**				**Comparability**		**Outcome**			**Total**
	1)	2)	3)	4)	a)	b)	1)	2)	3)	
**Scale: PC-ASPECTS per score decrease**										
Junji Uno 2020	*	*	*	*		*	*	*	*	9
Xuelei Zhang 2019	*	*	*	*		*	*	*	*	8
Sheng-Feng Lin 2018	*	*	*	*		*	*	*	*	8
Chuanhui Li 2018	*	*	*	*		*	*	*	*	8
Woong Yoon 2015	*	*	*	*	*	*	*	*	*	9
Isabelle Mourand 2014	*	*	*	*		*	*	*	*	8
Simon Nagel 2012	*	*	*	*		*	*	*	*	8
Hideaki Tei 2010	*	*	*	*		*	*	*	*	8

Each star (*) indicates one point of the scale

Cohort study (score 0 to 9; “high-quality”=studies with 6 or more stars)

Note= a) Comparability (point A) was tested whether the final functional outcomes were adjusted baseline characteristics of age and gender or not.

b) Comparability (point B) was tested whether the imaging modality was consistent in groups of each study
