# Supplementary material for: Posterior circulation acute stroke prognosis early CT scores in predicting functional outcomes: A meta-analysis
Source: PLoS One. 2021 Feb 16;16(2):e0246906. doi: 10.1371/journal.pone.0246906 (PMC7886215; doi:10.1371/journal.pone.0246906)
Supplement: S3 Table — (PDF) [file pone.0246906.s010.pdf]

S3 Table. Meta-regression of standardized mean difference of PC-ASPECTS between unfavorable and favorable outcomes for patients with intra-arterial endovascular treatment.

**Variables**	**Coef.**	**SE**	**95% CI**	***t test***	***P* value**
Constant	0.319	0.946	-1.789 to 2.427	0.34	0.743
Imaging modality of CT vs. MRI	-0.903	0.405	-1.805 to -0.002	-2.23	0.050*
Definition of UFO by mRS (3-6 vs. 4-6)	0.331	0.396	-0.551 to 1.213	0.84	0.422
REML estimate of between-study variance			$Tau^2 = 0.304$		
Number of observation= 13			Residual variation $I^2 = 71.01\%$		
			Adjusted $R^2 = 38.91\%$		

Coef., coefficient; CT, computed tomography; MRI, magnetic resonance imaging ; mRS, modified Rankin Scale; PC-APSECTS, posterior circulation Alberta stroke program early CT score; REML, Restricted maximum likelihood; SE, standard error; UFO, unfavorable function outcome
