# Supplementary material for: Posterior circulation acute stroke prognosis early CT scores in predicting functional outcomes: A meta-analysis
Source: PLoS One. 2021 Feb 16;16(2):e0246906. doi: 10.1371/journal.pone.0246906 (PMC7886215; doi:10.1371/journal.pone.0246906)
Supplement: S4 Table — (PDF) [file pone.0246906.s011.pdf]

S4 Table. Significant test for effect size for each cut-off of PC-ASPECTS

Cut-off point	z-score	Raw *P* value	Stepdown Bonferroni *P* value	Hochberg adjustment
PC-ASPECTS ≤ 6	2.45	0.0140	0.0210	0.0140
PC-ASPECTS ≤ 7	3.94	<0.0001	< 0.0001	<0.0001
PC-ASPECTS ≤ 8	2.72	0.0070	0.0210	0.0140
PC-ASPECTS ≤ 9	2.72	0.0070	0.0210	0.0140

PC-APSECTS, posterior circulation Alberta stroke program early CT score
