# Supplementary material for: Posterior circulation acute stroke prognosis early CT scores in predicting functional outcomes: A meta-analysis
Source: PLoS One. 2021 Feb 16;16(2):e0246906. doi: 10.1371/journal.pone.0246906 (PMC7886215; doi:10.1371/journal.pone.0246906)
Supplement: S1 File — (PDF) [file pone.0246906.s012.pdf]

The codes were listed in the following.

```
*SMD part*

label var mean1 "Unfavorable "
label var mean2 "Favorable "

metan n1 mean1 sd1 n2 mean2 sd2, lcols(Author Year mean1 mean2) textsize(150)  xlabel (-
1.5,-1,-0.5,0,0.5,1,1.5)  ///
title("PC-ASPECTS score difference between unfavorable and favorable outcome groups")

*CT vs MRI*
metan n1 mean1 sd1 n2 mean2 sd2, lcols(Author Year mean1 mean2) textsize(120)
by(imaging_modality) ///
xlabel (-1.5,-1,-0.5,0,0.5,1,1.5)   ///
title("PC-ASPECTS score difference between unfavorable and favorable outcome groups")

*mRS 2 vs 3*
metan n1 mean1 sd1 n2 mean2 sd2, lcols(Author Year mean1 mean2) textsize(120) by(mRS)
xlabel (-1.5,-1,-0.5,0,0.5,1,1.5)  ///
 title("PC-ASPECTS score difference between unfavorable and favorable outcome groups")


*OR part*
gen score = ""
replace score = "9" if PCASPECTS==9
replace score = "8" if PCASPECTS==8
replace score = "7" if PCASPECTS==7
replace score = "6" if PCASPECTS==6
assert score !=""
label var score "PC-ASPECTS"
tab score

gen lnOR = ln(OR)
gen lnORLL = ln(ORLL)
gen lnORUL = ln(ORUL)

generate se = (ORUL - ORLL) / (2*invnormal(0.975))
generate lnse = (lnORUL - lnORLL) / (2*invnormal(0.975))
generate varlnse = lnse^2
generate period = PCASPECTS
generate OR2 = exp(lnOR)

label define PCASPECTSlab 6 "PCASPECTS<=6" 7 "PCASPECTS<=7" 8 "PCASPECTS<=8" 9
"PCASPECTS<=9" , replace
label values PCASPECTS PCASPECTSlab
tab PCASPECTS
```

Metan: meta-analysis command

```
sort PCASPECTS Year
metan lnOR lnORLL lnORUL, random lcols(Author Year) eform effect(OR) by(PCASPECTS)
label(namevar=PCASPECTS) ///
xlabel(0.1,0.2,0.5,1,2,5,10) ///
title("Unfavorable outcomes prediction by binary PC-ASPECTS") ///
boxsca(50) xsize(10) ysize(8)
```

Metan: CT vs MRI

```
sort PCASPECTS Year
metan lnOR lnORLL lnORUL, random lcols(Author Year) eform effect(OR)
by(imaging_modality) label(namevar=PCASPECTS) ///
xlabel(0.1,0.2,0.5,1,2,5,10) textsize(150) ///
title("Unfavorable outcomes prediction by binary PC-ASPECTS") ///
boxsca(50) xsize(10) ysize(8)
```

Metan: mRS 2 vs 3

```
metan lnOR lnORLL lnORUL, random lcols(Author Year) eform effect(OR) by(mRS)
label(namevar=PCASPECTS) ///
xlabel(0.1,0.2,0.5,1,2,5,10) ///
textsize(150) title("Unfavorable outcomes prediction by binary PC-ASPECTS") ///
boxsca(50) xsize(10) ysize(8)
```

Metafunnel

```
metafunnel lnOR lnse, xtitle(Log Odds Ratio) subtitle() ///
ytitle(Standard error of Log Odds Ratio) by(PCASPECTS)
```

OR par score change

```
gen lnOR = ln(OR)
gen lnORLL = ln(ORLL)
gen lnORUL = ln(ORUL)
generate lnse = (lnORUL - lnORLL) / (2*invnormal(0.975))
generate varlnse = lnse^2


metan lnOR lnORLL lnORUL, random lcols(Author Year) eform effect(OR) nosecsub  ///
xlabel(0.1,0.2,0.5,1,2,5,10) textsize(150) title("Unfavorable outcome prediction by PC-
ASPECTS per score decrease") ///
boxsca(50) xsize(10) ysize(8)

metan lnOR lnORLL lnORUL, random lcols(Author Year) eform effect(OR)
by(imaging_modality) nosecsub  ///
```

```
xlabel(0.1,0.2,0.5,1,2,5,10) textsize(150) title("Unfavorable outcome prediction by PC-
ASPECTS per score decrease") ///
boxsca(50) xsize(10) ysize(8)

metan lnOR lnORLL lnORUL, random lcols(Author Year) eform effect(OR) by(mRS) nosecsub
///
xlabel(0.1,0.2,0.5,1,2,5,10) textsize(150) title("Unfavorable outcome prediction by PC-
ASPECTS per score decrease") ///
boxsca(50) xsize(10) ysize(8)

*metafunnel

metafunnel lnOR lnse, xtitle(Log odds ratio) ytitle(Standard error of log OR)
confunnel lnOR lnse

*metabias

metabias lnOR lnse, begg
```
